# Lactotransferrin promotes intervertebral disc degeneration by regulating Fas and inhibiting human nucleus pulposus cell apoptosis

**DOI:** 10.18632/aging.204100

**Published:** 2022-05-25

**Authors:** Xiao-Bo Zhang, Si-Qi Xu, Yi-Geng Hui, Hai-Yu Zhou, Yi-Cun Hu, Rui-Hao Zhang, Xi-Dan Gao, Chang-Ming Zheng

**Affiliations:** 1 Department of Spine Surgery, Honghui Hospital, Xi'an, Shanxi 710000, PR China; 2 Department of Spine Surgery, Lanzhou University Second Hospital, Lanzhou, Gansu 730000, PR China

**Keywords:** lactotransferrin, intervertebral disc degeneration, bioinformatics, nucleus pulposus cell, mechanism

## Abstract

Background: In recent years, intervertebral disc (IVD) degeneration (IDD) has increased in age. There is still a lack of effective treatment in clinics, which cannot improve the condition of IDD at the level of etiology.

Objective: To explore IDD pathogenesis at the cellular and gene levels and investigate lactotransferrin (LTF) expression in IDD patients and its possible mechanism.

Methods: We downloaded the IDD data set from the Gene Expression Omnibus (GEO) database, screened the differentially expressed genes (DEGs) and hub genes and performed Kyoto Encyclopedia of Genes and Genomes (KEGG) analysis to construct a protein–protein interaction (PPI) network. Subsequently, we verified LTF's regulatory mechanism through cell experiments. IL-1β was used to intervene in nucleus pulposus cells (NPCs) to construct the IDD cell model, and LTF and Fas expression was detected by qRT–PCR. LTF inhibitor, Fas inhibitor, LTF mimic, and Fas mimic were used to intervene in each group. Western blotting was used to detect Fas, Caspase-3, Bax, and Bcl-2 expression.

Results: A total of 131 DEGs and 10 hub genes were screened. LTF mRNA in the IDD model was significantly higher than that in the control group, while Fas' mRNA was significantly lower. When LTF was upregulated or downregulated in NPCs, apoptosis marker expression showed the opposite trend. The rescue test showed that LTF and Fas' overexpression greatly enhanced NPC apoptosis.

Conclusion: LTF promotes IDD progression by regulating Fas in NPCs, and it may be an effective gene therapy target.

## INTRODUCTION

In the past 200 years, low back pain has been one of the most common musculoskeletal diseases affecting medical resource consumption worldwide [1]. Among them, IDD is considered a widely recognized cause of low back pain. It is a chronic degenerative spinal disease that often leads to back, neck, and nerve root pain, the leading cause of global disability [2–4]. It will cause nerve tissue compression and pain due to spinal stenosis in the long run. With the increase in the elderly population, this problem has become increasingly severe [5]. It makes the patient lose the ability to work and causes a significant economic burden to the family and society [6, 7]. Although scholars have conducted in-depth research on IDD in recent decades, the specific pathogenesis is still unclear. It is a complex multifactorial process that has been proven to be related to biomechanical factors [8, 9], apoptosis-related theory [6], inflammatory factor destruction theory [10], and genetic factors [11]. In recent years, scholars have gradually deepened the research on the role of apoptosis in IDD. Classical apoptosis pathways mainly include the Fas death receptor pathway, mitochondrial pathway, and endoplasmic reticulum stress pathway [12]. The Bcl-2 protein and the caspase families are currently the most concerning among the many apoptosis-regulating genes.

The current treatments for IDD are limited and cannot be cured on the etiological level. Many patients finally choose surgical treatment, but surgical treatment is traumatic, expensive, and sometimes has some complications, which brings a huge economic burden to the family and society. Finding precise gene-targeted therapy is the direction of future development. With the rapid development of gene expression microarray technology, it is possible to use bioinformatics methods for data mining and analysis [13, 14]. It can reveal the abnormally expressed genes in degenerative tissues and predict their possible mechanism. By analyzing differential expression genes (DEGs), we screened the most significant 10 Hub genes and found that the lactotransferrin (LTF) expression was significantly upregulated in IDD samples. Thus far, the LTF’s role in IDD has not been reported. We found that IDD’s pathogenesis involves apoptotic pathways through enrichment analysis of the gene set variation analysis (GSVA) Hallmark pathway. Previous studies have shown that activating the apoptotic pathway will lead to IVD cell loss and extracellular matrix (ECM) degradation and accelerate the disease’s deterioration [15]. NPCs apoptosis plays an important role in the pathogenesis of IDD, while LTF plays a special regulatory role in cells mainly by regulating apoptosis. However, there is no sufficient evidence to explain the relationship between LTF and nucleus pulposus (NP) cells (NPCs) apoptosis. Therefore, the purpose of this study is to prove whether LTF induces the occurrence of IDD by regulating apoptosis, which is of great significance for further exploring the etiology of IDD, achieving precise treatment of the disease.

## MATERIALS AND METHODS

### Data download and preprocessing

The GEO query package was used to download the gene expression profile datasets GSE124272 and GSE153761 from the GEO database. The GSE124272 dataset includes eight patients with IDD diagnosed by magnetic resonance imaging and eight volunteers with IVD samples without IDD clinical evidence, such as low back pain sciatica. The GSE153761 dataset includes three degenerative patients and three normal patients with IVD samples. The original data were read via the Affy package [16]. The original CEL files were subjected to background correction and data normalization through the RMA algorithm. The correction effect between samples uses two-dimensional principal component aggregation. The principal component analysis (PCA) diagram is displayed, and then the gene annotation file GPL570 platform is used to annotate the expression matrix [17].

### DEGs screening and pathway enrichment analysis

After data preprocessing, DEGs were screened through the limma package [18], and |log2-fold change (log2FC)| > 1 and *P* < 0.05 were used as the screening criteria. Then, the ggplot2 package was used to draw a volcano map to show the DEGs’ differential expression.

The Kyoto Encyclopedia of Genes and Genomes (KEGG) is a database resource that integrates genomics, chemistry, and system function information, providing known biological metabolic signaling pathways [19]. In addition, we used the cluster profile package [20] to analyze DEG KEGG pathway enrichment. *P* < 0.05 was considered significant enrichment.

### Construction of a protein–protein interaction (PPI) network and screening Hub genes

The Search Tool for the Retrieval of Interacting Genes (STRING) (http://string-db.org; version: 11.0) [21] is an online tool for evaluating PPI information. DEGs were imported into STRING online software to obtain the PPI relationship and exported in TSV format. Then, the obtained source file was imported into Cytoscape software [22], and the plug-in NetworkAnalyzer [23] and cytoHubba [24] were used to screen Hub genes. The top 10 scores are selected as Hub genes using the MCC algorithm. Based on the semantic similarity of the GO terms of gene annotation [25], we used the GOSemSim package [26] to calculate the strength of the relationship between the molecular functions and cell positioning of 10 Hub genes. We ranked them by the average value of functional similarity. The hub genes were sorted with a cutoff value of 0.7, and the results were visualized by the cluster profile package [20].

### GSVA analysis

To reveal the difference in signal pathway enrichment between IDD samples and control samples, we used the GSVA package [27] (https://github.com/rcastelo/GSVA) to evaluate the t value and assign pathway activity conditions and used the ggplot2 package [28] to plot and display the significant results.

### NPC extraction and primary culture

The ethics committee approved the Lanzhou University Second Hospital study for extracting NPCs from NP tissues of young patients (patients under 25 years of age). MRI was used as the observation index of IDD, and grade II was used as the control group. After washing the NP specimen three times with PBS, the NP tissue was cut into 1 mm³ pieces. Then, we used 0.25% pancreatin and 0.2% collagenase for digestion. Finally, the samples were filtered with a 70 μm cell sieve and centrifuged. The supernatant was discarded, and the cells were inoculated into a 6-well plate. After the cells adhered to the wall, we changed the solution every 2 to 3 days.

### Toluidine blue staining and safranin O-fast green staining

We dyed according to the toluidine blue staining instructions (1% phosphate method). NPCs were seeded on cover glass at a density of 1 × 105/min and cultured in a cell incubator. When the cells grew approximately 80%, they were washed with PBS, fixed with 95% alcohol, and washed three times. Toluidine blue staining solution was added to the dye.

Similarly, we dyed according to the modified Safranin O-fast green staining instructions. After washing with PBS, NPCs were fixed with 4% paraformaldehyde. Then, the NPCs were stained with hematoxylin before they were washed three times with PBS and finally stained with dye solution.

### Construction of the IDD cell model

According to previous experimental studies, we treated NPCs with IL-1β at a concentration of 10 ng/mL for 24 hours and then extracted RNA from NPCs.

### Cell transfection

The cell transfection in this study included LTF mimic, LTF negative control (NC), LTF inhibitor, anti-LTF negative control (inhibitor NC), si-Fas, and siRNA control (si NC) (Shanghai Gene Pharmaceutical Company, China). The Fas sequence was synthesized and cloned into the transfection plasmid to obtain the recombinant vector pcDNA3.1-Fas. According to the instructions, all mRNA analogs, inhibitors, and Fas constructs were transiently transfected into cells for 48 h using Lipofectamine 2000 (Invitrogen, USA).

### qRT–PCR

According to the manufacturer’s instructions, total RNA was extracted from NPCs with TRIzol reagent (Invitrogen) and then reverse transcribed and amplified by qRT–PCR. The primers for qRT–PCR are shown in Table 1.

**Table 1:** Primer sequences.

**Gene**	**Primer**	
*LTF*	*forward*	5′-CGCGATCCCACCACTGC-3′
	*reverse*	5′-AGTGCAGGGTCCGAGGTATT-3′
*Fas*	*forward*	5′-AGCCACAGGCACCTTGAGGAC-3′
	*reverse*	5′-GTACTGCTGGTGGATGTCGTTCAG-3′
*β-actin*	*forward*	5′-AATGGGCAGCCGTTAGGAAA-3′
	*reverse*	5′-GCGCCCAATACGACCAAATC-3′

### Western blot

According to the standard protocol, the total protein was separated using a protein extraction kit (Sell Chemical Company). The antibodies used were LTF (1:100), Fas (1:100), Bax (1:1000), Bcl-2 (1:1000), Caspase-3 (1:1000) and β-actin (1:1000). We used ECL luminescent solution to expose and develop color in the gel imaging system.

### Statistical analysis

All data were statistically processed by R software, and the measurement data are expressed as the mean ± standard deviation. One-way ANOVA was performed. The LSD test was used for homogeneous variance, and Dunnett’s t multiple tests were used for uneven variance after correction. *P* < 0.05 indicates that the difference is statistically significant.

### Research involving human and animal participants

This experiment was approved by the ethics committee of Lanzhou University Second Hospital.

## RESULTS

### Bioinformatics analysis

**Figure 1:**
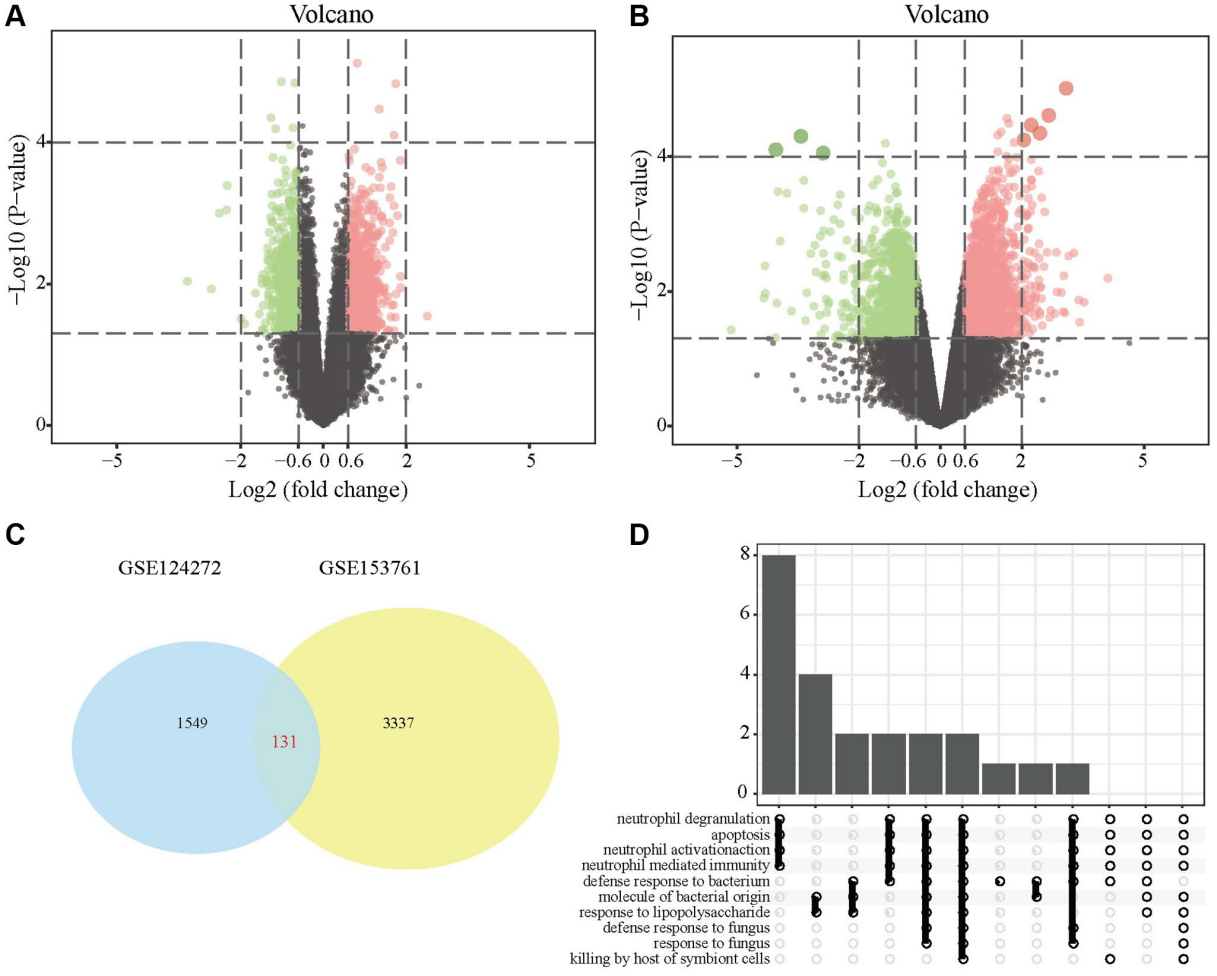
**DEGs in IDD tissues and normal tissues.** (**A**) DEG volcano map in the GSE124272 data set; (**B**) DEG volcano map in the GSE124272 data set; (**C**) Venn diagram of the intersection of the DEGs of the two data sets GSE124272 and GSE153761. (**D**) Enrichment analysis of the KEGG signaling pathways of DEGs.

First, we conducted DEG screening and pathway enrichment analysis. After data preprocessing, we used R software to analyze the difference between the two data sets GSE124272 and GSE153761 and obtained 1549 and 3337 DEGs. The results are presented as volcano plots (Figure 1A, 1B). The DEGs obtained in the two data sets were intersected, and the overlapping part contained 131 DEGs, as shown in the Venn diagram (Figure 1C). KEGG analysis showed that the DEG enrichment pathways were mainly involved in neutrophil degeneration, apoptosis, neutrophil activation, immune response mediated by medium- and fine-grained cells, and lipopolysaccharide response (Figure 1D).

**Figure 2:**
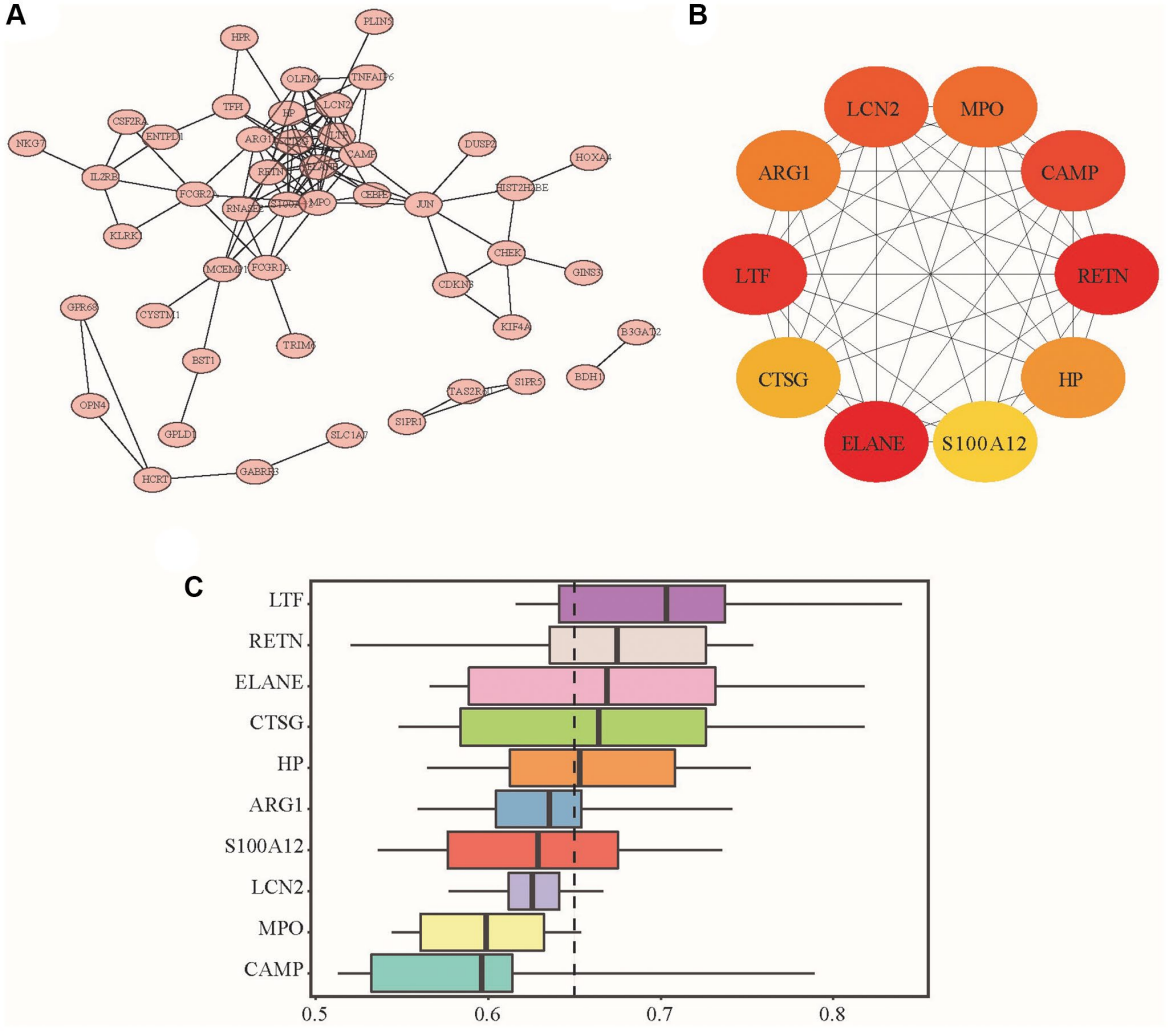
**PPI network, hub genes and DEG functional similarity scores.** (**A**) PPI network analysis diagram; (**B**) Hub gene schematic diagram: the redder the color is, the higher the enrichment score is, and the yellow represents the relatively small enrichment score. (**C**) The distribution of the functional similarity of different genes is summarized as a box plot. The middle of these boxes represents 50% similarities; the upper and lower borders show the 75th and 25th percentiles. The line in the box represents the average value of functional similarity.

Second, we used STRING to construct the DEG PPI network and used Cytoscape to visualize it. Figure 2A shows the interaction between DEGs, where the network of interactions between LTF and other DEGs was relatively concentrated. Moreover, we used the cytoHubba plug-in to select the top 10 genes in the most prominent relevant criteria as Hub genes, namely, ELANE, RETN, LTF, CAMP, LCN2, MPO, ARG1, HP, CTGF and S100A12 (Figure 2B, 2C). According to the MCC algorithm, the DEG scores obtained are shown in Table 2. The top three scores were ELANE, RETN, and LTF, suggesting that they were more likely to be critical genes of IDD. We repeatedly verified their differential expression in the control and IDD groups through qRT–PCR. We finally found that ELANE and RETN were not significantly different between the two groups, so we chose LTF as our target gene.

**Table 2:** 10 Hub genes.

**Rank**	**Gene Name**	**Full Name**	**Score**
1	*ELANE*	Elastase, Neutrophil Expressed	25478
2	*RETN*	Resistin	25446
**3**	***LTF***	**Lactotransferrin**	**25248**
4	*CAMP*	Cathelicidin Antimicrobial Peptide	25230
5	*LCN2*	Lipocalin 2	25201
6	*MPO*	Myeloperoxidase	20446
7	*ARG1*	Arginase 1	15242
8	*HP*	Haptoglobin	15145
9	*CTSG*	Amphiphysin	10346
10	*S100A12*	S100 Calcium Binding Protein A12	10212

**Figure 3:**
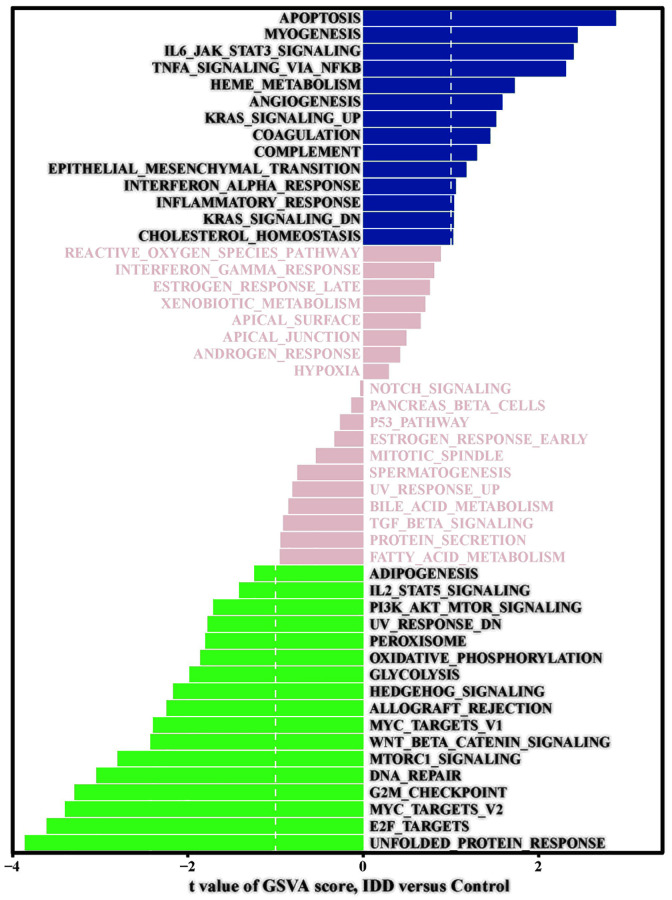
**GSVA signal path analysis.** GSVA score between IDD and normal group samples; t value is displayed from the linear model, we set |t|>1 as the cutoff value.

Finally, we performed GSVA to explore the important functional pathways between the IDD and normal group samples. Pathway enrichment analysis showed (Figure 3) that the apoptosis pathway, IL6-JAK-STAT3 signaling pathway, angiogenesis pathway, KRAS signaling pathway, and complement system signaling pathway were significantly activated. In contrast, the E2F target, MYC target, G2/M checkpoint pathway, DNA repair, Wnt/β-Catenin signaling pathway, Hedgehog signaling pathway, glycolysis, and oxidative phosphorylation signaling pathway were significantly inhibited.

### LTF and Fas expression in NPCs

**Figure 4:**
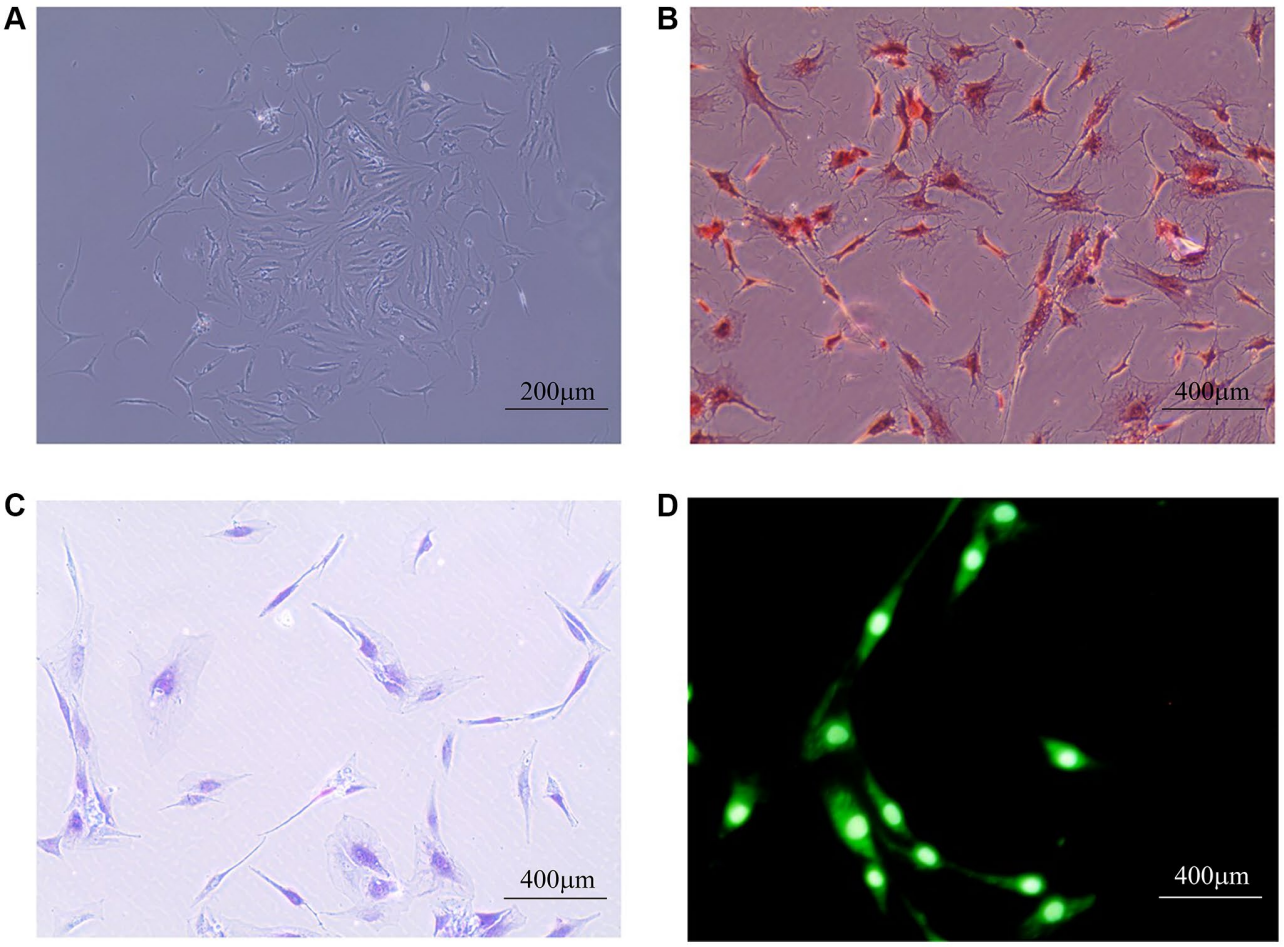
**Human primary NPC identification.** (**A**) The general morphology of primary NPCs, which mainly were star-shaped, long fusiform, polygonal or irregular under the microscope; (**B**) Safranin-O fast green staining: NPCs nucleus is stained dark red, cytoplasm is lightly stained; (**C**) Toluidine blue staining: NPCs were dyed indigo blue, and the nucleus is located in the center of the cell or tilted to one side; (**D**) Immunofluorescence staining: The fluorescence of type II collagen is widely expressed in NPCs, most of which are located in the cytoplasm. The closer to the nucleus, the stronger the fluorescence intensity.

**Figure 5:**
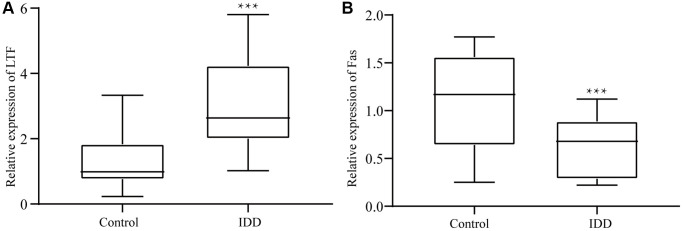
In the IDD group, (**A**) LTF expression was significantly downregulated, (**B**) while Fas expression was significantly upregulated. ^***^P < 0.001.

As shown in Figure 4, we identified the extracted cells and determined them to be NPCs for follow-up experiments. To explore the roles of LTF and Fas in IDD, we used qRT–PCR to compare LTF and Fas’ expression in the control and IDD groups. The results showed that LTF expression was significantly higher than that in the control group (Figure 5A). Simultaneously, Fas was significantly lower (Figure 5B).

### LTF inhibited NPC apoptosis and Fas expression

**Figure 6:**
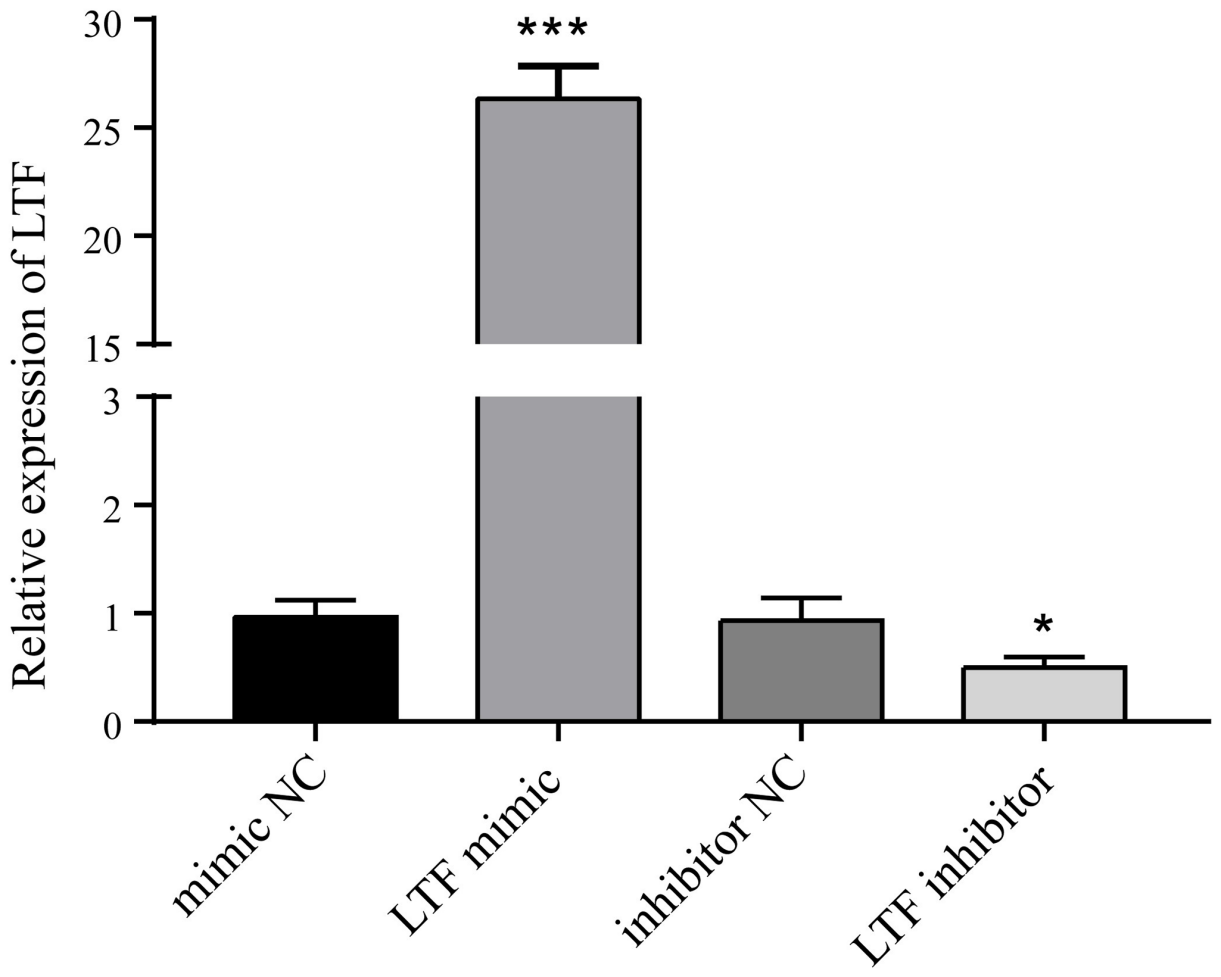
**LTF activity after LTF mimic and inhibitor transfection.**^ *^P < 0.05 and ^***^P < 0.001.

**Figure 7:**
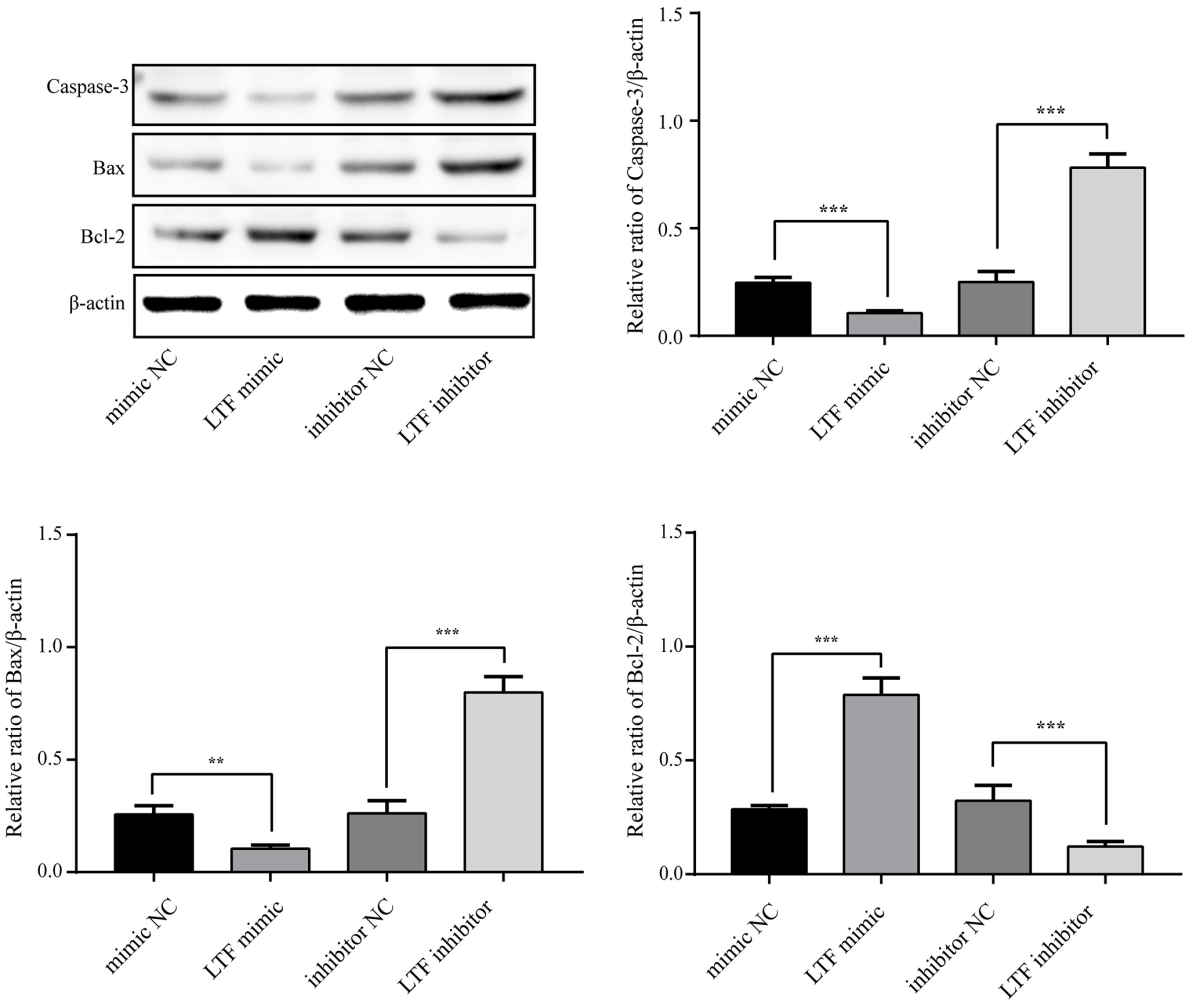
**LTF inhibited NPC apoptosis.**^ **^P < 0.01 and ^***^P < 0.001.

To clarify whether LTF affects cell apoptosis, we used LTF mimics and inhibitors and used qRT–PCR to detect LTF expression in NPCs. As expected, compared with the NC group, the LTF activity was significantly increased in the cells transfected with LTF mimic, while the results of the LTF inhibitor transfection were the opposite (Figure 6). Western blot results showed that LTF significantly inhibited NPC apoptosis, while decreased LTF activity significantly reversed this effect (Figure 7).

**Figure 8:**
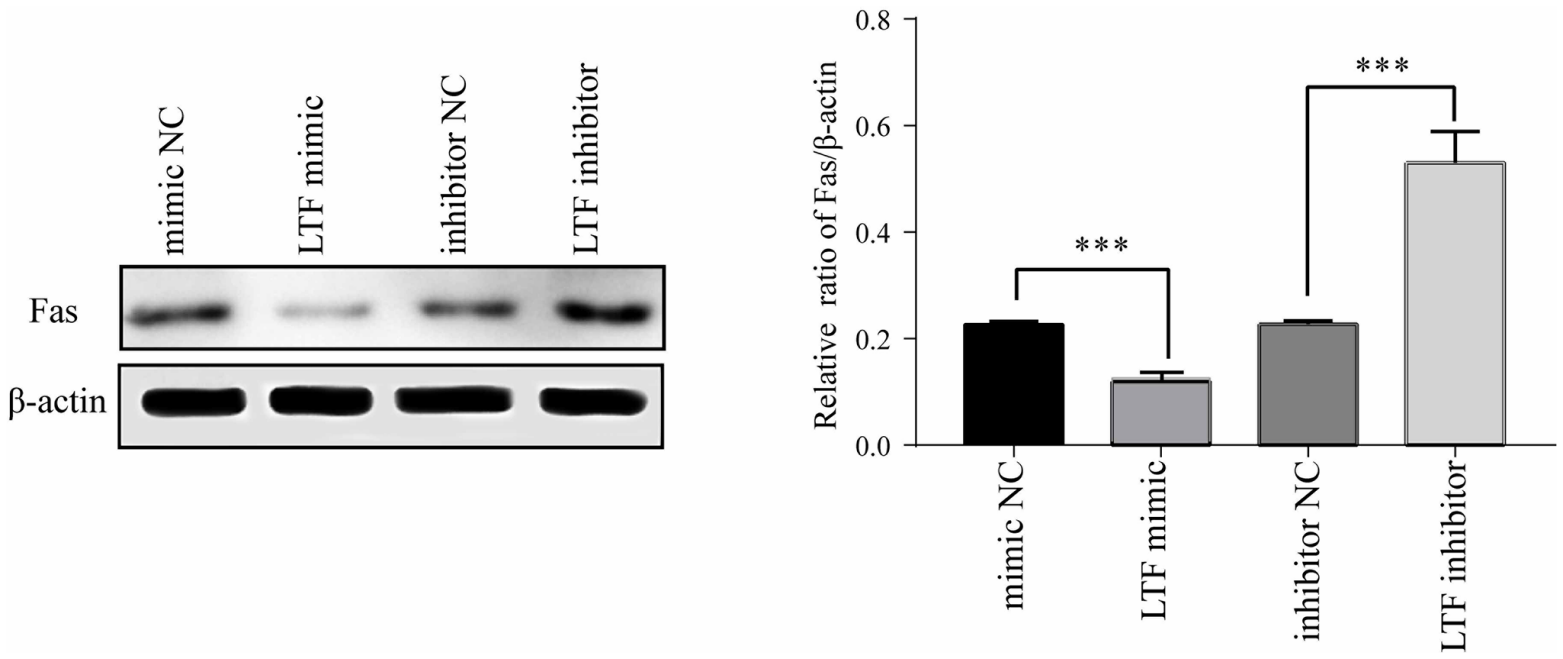
**Fas expression in NPCs after transfection with LTF mimic and LTF inhibitor.**^ *^P < 0.05 and ^***^P < 0.001.

Fas expression was significantly reduced after transfection with the LTF mimic, and at the same time, its protein expression was significantly increased after transfection with the LTF inhibitor (Figure 8). These results showed that LTF directly targets Fas in NPCs and has an inhibitory effect.

### LTF inhibited NPC apoptosis by targeting Fas

**Figure 9:**
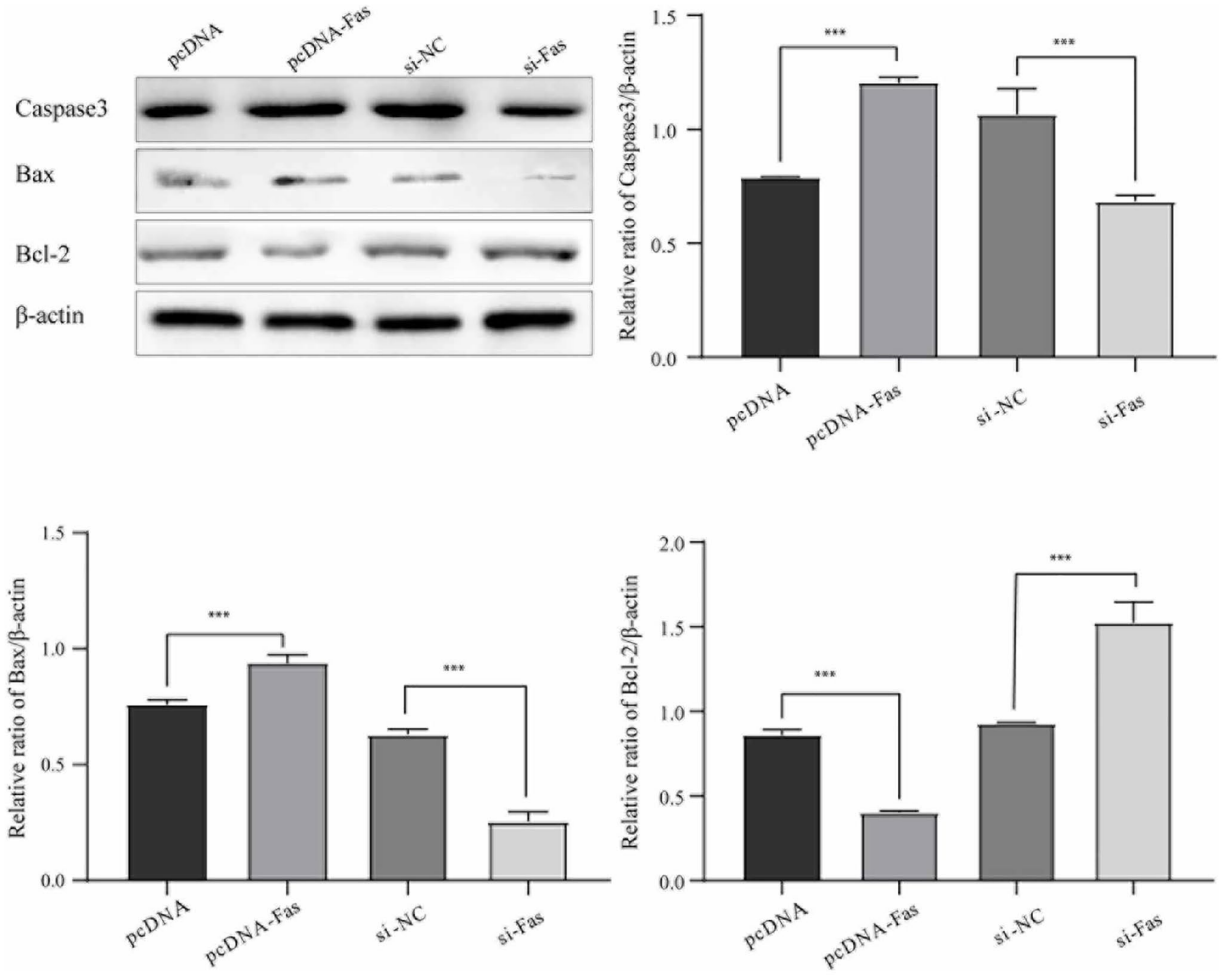
**Fas promoted NPC apoptosis.**^ *^P < 0.05 and ^**^P < 0.01.

Studies have reported that Fas is abnormally activated in IDD. To understand Fas’s role in NPCs, we further analyzed the effect on cell apoptosis. As shown in Figure 9, Fas’ overexpression significantly promoted cell apoptosis. Knocking out Fas can dramatically reduce cell apoptosis, indicating that Fas plays an essential role in NPCs.

**Figure 10:**
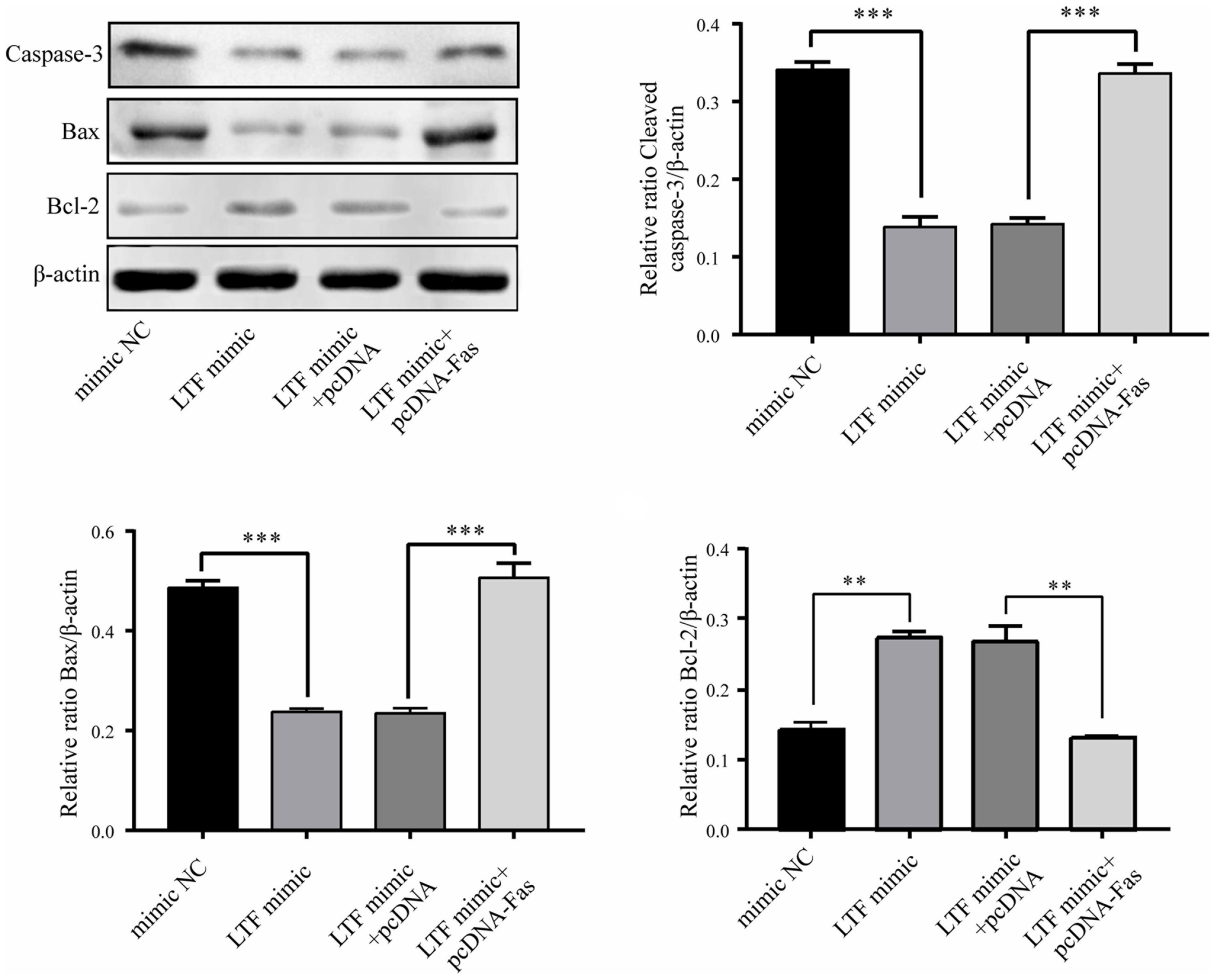
**LTF inhibited apoptosis by targeting Fas in NPCs.**^ **^P < 0.01, ^***^P < 0.001.

To explore whether Fas is involved in the biological role of LTF in NPCs, pcDNA-Fas was used to transfect LTF-overexpressing NPCs. The results showed that LTF and Fas overexpression promoted apoptosis (Figure 10). The above results further indicated that LTF inhibits apoptosis by targeting Fas.

## DISCUSSION

This study analyzed IDD DEGs through bioinformatics technology and selected LTF as our target gene. Through *in vitro* cell experiments, we found that LTF was significantly upregulated in IDD tissues, which was in line with the results of bioinformatics analysis. Subsequently, based on the results of the GSVA, we speculated that LTF might induce IDD by regulating apoptosis. The relationship between LTF and classical apoptotic molecules was verified by western blot. We believe that LTF may be an essential factor in inducing IDD related to apoptosis.

LTF is an 80 kDa basic glycoprotein with iron-binding properties. It exists in most body fluids and is widely expressed in milk, trachea, saliva, nasal secretions, and neutrophil granules [29]. It has anti-inflammatory, antibacterial, and antitumor properties and regulates cell growth, differentiation, and iron transport in mammals. These properties of LTF give it great therapeutic potential [30]. Additionally, LTF is a potent inhibitor of eosinophil migration and may be an effective therapeutic agent to control eosinophil infiltration [30]. It can also initiate an immune cascade to increase cytokine release (IL-18) and cell activity (cytotoxic T cells). After oral administration, LTF shows high bioavailability, high selectivity for cancer cells, and multiple molecular targets that control tumor proliferation, survival, migration, invasion, and metastasis. It is a potential anticancer drug choice [31]. Of note, LTF can promote or inhibit cell proliferation and migration depending on whether it acts upon normal or cancerous cells, respectively [31]. In addition, it is regarded as the first-line mediator of immune defense and response to pathogenic and nonpathogenic damage. It can be used alone or as an adjuvant against a variety of antibiotic-resistant bacteria and other pathogens. It is an excellent natural substitute [32].

LTF is related to apoptosis, and it can play a role by promoting apoptosis [33, 34]. For example, lactoferrin B-like peptide exerts its antibacterial activity by inducing apoptosis-like death [35]. At the same time, it can also inhibit apoptosis [36, 37], especially in aging-related diseases, such as Parkinson’s disease [38], which may depend on the type of protein that binds to it. LTF can protect mesenchymal stem cells from senescence and apoptosis caused by oxidative stress [37]. However, to date, there have been no related reports on the role of LTF in IDD. In short, we verified LTF’s negative regulatory effect on NPC apoptosis for the first time. We confirmed that this effect might be achieved by acting on Fas, guiding the significance of IDD targeted therapy. However, Fujita et al. [39] found that LTF enhances Fas expression and apoptosis in the colon mucosa of azoxymethane-treated rats, which is inconsistent with our research results. At the same time, some scholars have found that LTF exerts its protective effect on chondrocytes by inhibiting apoptosis (such as caspase-3 and Fas) [40]. At present, LTF has been shown to have a preventive effect on diseases in many fields, but its mechanism against different diseases is mainly related to apoptosis. Under different conditions, why the regulation of apoptosis by LTF shows the opposite regulatory mechanism needs further exploration and in-depth research.

Unlike previous studies, our research suggested that LTF promotes IDD by inhibiting apoptosis rather than promoting apoptosis. IDD is an age-related disease. Under the influence of various external factors, the continuous activation of the apoptotic pathway may be essential in causing IDD. While we used NP tissue from young patients with early degeneration, there may be individual differences. Second, there may be a specific compensatory mechanism in the early phase of the disease, or the experiment triggers a specific protective mechanism inside the cell. Of course, this requires further verification by experiments. In addition, LTF also has a particular anti-inflammatory effect, and inflammation is also one of the crucial causes of IDD. Whether LTF affects the progression of IDD by regulating the inflammatory response remains to be studied.

Although we used cell experiments to verify the results of the bioinformatics analysis, there are still the following limitations. First, the data set selected in this study has a certain degree of heterogeneity. Although we carried out quality control and standardization on the original data, we still need a larger sample size and higher quality data set to verify this study's reliability. Second, this study only conducted experimental verification at the cellular level and did not conduct body-level research, and the apoptosis indicators are not comprehensive. For IVD, IVD cell regeneration and the ECM increase may be effective indicators for IDD treatment. We have not verified this. Finally, due to time constraints, we did not exclude the time-dependent factors of cells.

## CONCLUSIONS

LTF is significantly upregulated in degenerative IVD tissues, and LTF improves IDD progression by inhibiting Fas’ expression. This study provides a theoretical basis for further research on the molecular mechanism of IDD and helps develop potential therapeutic targets.
